# Resourse Use and Disease Couse in dementia - Nursing Home (REDIC-NH), a longitudinal cohort study; design and patient characteristics at admission to Norwegian nursing homes

**DOI:** 10.1186/s12913-017-2289-x

**Published:** 2017-05-22

**Authors:** Irene Røen, Geir Selbæk, Øyvind Kirkevold, Knut Engedal, Ingelin Testad, Sverre Bergh

**Affiliations:** 1 Centre for Old Age Psychiatric Research, Innlandet Hospital Trust, Ottestad, Norway; 2 Norwegian National Advisory Unit on Ageing and Health, Vestfold Hospital Trust, Tønsberg, Norway; 3 Faculty of Medicine, University of Oslo, Oslo, Norway; 4 Norwegian University of Science and Technology (NTNU), Department of Health Science in Gjøvik, Gjøvik, Norway; 5 Centre for Age-Related Medicine, Stavanger University Hospital, Stavanger, Norway

**Keywords:** Nursing homes, Dementia, Neuropsychiatric symptoms, Resource use, Cohort-study, Longitudinal

## Abstract

**Background:**

Earlier studies of nursing home patients show a high prevalence of dementia, neuropsychiatric symptoms (NPS), pain, and dependency in activities of daily living. The REDIC-NH cohort was set up to study the disease course and the resources used in patients with dementia in Norway. The aim of this paper was to describe the methods and the data collection, and to present selected data about patients at admission to a nursing home.

**Methods:**

We included 696 patients at admission to a nursing home and followed them with biannual assessments until death. Baseline data were collected between March 2012 and November 2014. In October 2016, patients had either completed an 18-month follow-up (*n* = 349), passed 18 months without assessments (*n* = 22), or left the study (*n* = 324). Data on demographics, cognition, NPS, activities of daily living (ADL) functioning, physical health, medication, Quality of Life (QoL), resource use, and caregiver burden, in addition to DNA samples were collected.

**Results:**

Mean age of the participants at inclusion was 84.5 years (SD 7.5, range 50 – 105), 63.9% were women. According to data collected in the study, 83.8% had dementia, but only 55.9% of them had a diagnosis of dementia registered in their records. The most frequent dementia diagnosis was Alzheimer’s disease, which was present in 71% of those with dementia. Patients with dementia more often experienced delusions, hallucinations, agitation, anxiety, disinhibition, irritability, and aberrant motor behaviour compared to patients without dementia. Depression and anxiety were the most common NPS symptoms.

**Conclusions:**

Dementia and NPS were highly prevalent among persons admitted to nursing homes. Only 55.9% of the patients with dementia had a diagnosis of dementia registered in their records.

## Background

Dementia is a syndrome caused by a variety of brain disorders, characterised by a decline in cognition, decreased ability to perform activities of daily living (ADL), and deterioration in emotional control, social behaviour, or motivation. The syndrome is usually of a chronic or progressive nature. Age-specific prevalence rates for dementia show an increase from 1.6% in the 60-64 age group to 21.7% in the 85-89 age group and to 43.1% in the 90+ age group [1]. Consequently, as the population ages, the number of persons with dementia is increasing worldwide, and is expected to double in the next 20 years [1]. A systematic review from 2013 reported that dementia is one of the most strongly associated factors to nursing home admission [2]. The prevalence of dementia in Norway in 2016 was estimated to be 78,000 [1], 1.5% of the total population. A Norwegian cross-sectional study showed that more than 80% of Norwegian nursing home (NH) patients had dementia defined with a Clinical Dementia Rating scale (CDR) score of 1 or above, and 72% of the patients with dementia had clinically significant neuropsychiatric symptoms (NPS) [3]. NPS include psychiatric symptoms such as delusions, hallucinations, depression, anxiety, and euphoria, and behavioural symptoms such as agitation, aggression, apathy, and disinhibition. According to a systematic review by Selbæk et al, NPS are common among patients with dementia, and the course of individual NPS varies considerably. Agitation (36%), apathy (36%), aggression (32%) and depression (28%) are the most prevalent symptoms [4]. Agitation and apathy are the most persistent NPS over time [4], and the prevalence of individual NPS changes with the progression of the dementia [2, 5–7]. Recent NPS studies have included genetic association designs, due to the strong familial aggregation of symptoms implicating genetic variation as a mediating factor [8]. Genetic polymorphism in serotonin and dopamine receptors have been found of importance both in the development of NPS as well as in treatment efficacy [9]. Psychotropic drugs are often used to treat NPS, despite uncertain efficacy and considerable risks for serious adverse events [10]. Data from randomised controlled trials and large registry-based studies indicate that the use of antipsychotic drugs is associated with increased mortality and an increased risk of cerebrovascular adverse events [11]. However, results from observational studies of clinical samples have been conflicting [12]. Depression is associated with increased mortality, but how antidepressant use and gender influence mortality is unclear [13–15]. Dementia leads to severe disability and causes a high burden on caregivers and costs to society. The economic burden in Europe has been estimated to be €55-66 billion annually [16]. The cost of dementia in Norway is estimated to be €3.02 billion a year [17].

Previous NH studies from Scandinavian have been cross-sectional [3, 18], but few studies have examined the course of dementia symptoms and the use of resources, from admission to NHs until death [5]. Only one grey paper in Norwegian has presented numbers for resource use in dementia in Norway [17], and a review of international studies has reported a large variation in cost estimates [16]. Previous studies have shown that more than 80% of the patients in Norwegian NHs have dementia, but only about 50% of those with dementia receive a diagnose of dementia. Thus, we decided to include all patients above 65 years at admission to NHs, in addition to those under 65 years with a diagnose of dementia. The present study is the first reporting the prevalence of dementia at admission.

The aim of the REDIC-NH study is to follow long-term NH patients from admission to the NH and until death. The study is designed to collect broad information to describe the course of dementia and other psychiatric and somatic diseases in NH patients from admission until death. These data will be used in several studies. More specifically:

- To describe the course of dementia in NH patients from admission until death.
- To identify predictors of progression of dementia in NH patients, with a particular focus on predictors of the course of neuropsychiatric symptoms.
- To investigate predictors of mortality in NH patients.
- To explore the impact of genetic polymorphism on the occurrence and course of neuropsychiatric symptoms in dementia.
- To study health resource use at admission and over the follow-up period.

In this paper, we describe the methods and the data collection in the REDIC-NH study. We will also present demographic data and data on dementia and NPS from the patients at admission to NHs.

## Methods

### Study design and setting

The REDIC-NH study was an observational longitudinal study including patients from a convenience sample of 47 NHs in four Norwegian counties, representing small and large NHs, located in urban and rural areas. Inclusion was at admission to the NH, and participants were followed until death. Due to substantial workloads, four NHs withdrew from the study during the study period.

The baseline data were collected within one month of admission to the NH. Baseline data were collected between March 2012 and November 2014. Follow-up data were collected every six months until the death of the patient, and were on-going.

### Participants

Participants were recruited at admission to the NH (*n* = 696). Patients eligible for inclusion in the study were 65 years or older, or younger than 65 years with established dementia, with an expected stay in the NH of more than four weeks. The only exclusion criterion was a life expectancy of less than six weeks.

### Data collection

The data collection was performed by healthcare workers in the NHs, mainly registered nurses (74%), under supervision of 10 research nurses. The research nurses completed a five-day training program, and the data collectors completed a two-day training program. Data were collected through structured interviews with the patient, their next of kin, and the caregivers in the NHs. Demographic data were collected through a review of patient documentation (see Table 1). DNA samples were obtained by collected saliva samples from the patients. The diagnosis of dementia was based on a review of data collected from the patients, their family members, and their caregivers after three physicians with ample clinical experience made a dementia diagnosis according to established criteria [19–22].

**Table 1:** Assessment instrument

	Description	Base-line version 1 0212 *n*=153	Base-line^a^ version 2 0712 *n*=391	Base-line^b^ version 3 0813 *n*=152	Follow up 6 mnd version 1 0812 *n*=318	Follow up^b^ 6 mnd version 2 0813 *n*=191	Follow up from 12 mnd version
Physical health status							
Blood pressure and pulse		x	x	x	x	x	x
Body Mass Index		x	x	x	x	x	x
General Medical Health Rating (GMHR) [31]	Four-category scale rating medical health	x	x	x	x	x	x
Mobilization-Observation-Behaviour-Intensity-Dementia Pain Scale (MOBID-2) (Including VAS) [32]	Assessment of pain in patients with dementia	x	x	x	x	x	x
Unified Parkinson’s Disease Rating Scale (UPDRS), six-item version [33]	Assessment of extra-pyramidal symptoms	x	x		x		
Edmonton Symptom Assessment System (ESAS-r) [34]	Assessment of pain and distressing symptoms such as fatigue, drowsiness, nausea, appetite disturbances, dyspnoea, depression, anxiety, and wellbeing		x	x			
Karnofsky Performance Status (KPS) [35]	Functional performance status		x	x	x		
Resident Assessment Instrument (RAI), subscales skin and nutrition [36]	Distressing symptoms, care and treatment provided		x		x		
Charlson’s co-morbidity index [37]	Co-morbid conditions	x	x	x	x		
Cognitive function and severity of dementia							
Mini Mental State Examination (MMSE) [23]	Screening for cognitive impairment	x	x	x	x	x	x
Severe Impairment Battery – 8 (SIB-8) [24]	Cognitive impairment in severe dementia	x	x	x	x	x	x
Informant Questionnaire on Cognitive Decline in the Elderly (IQCODE) [26]	Informant-rated scale of estimated cognitive decline	x					
Clinical Dementia Rating Scale (CDR) [28]	Level of dementia (cognition and function)	x	x	x	x	x	x
Functional Assessment Staging of Alzheimer’s Disease (FAST) [30]	Level of dementia (cognition and function)		x	x	x	x	x
Diagnoses	Type of dementia according to an algorithm	x	x	x			
Neuropsychiatric and depressive symptoms							
Neuropsychiatric Inventory Nursing Home version (NPI-NH) [38]	Neuropsychiatric symptoms	x	x	x	x	x	x
Neuropsychiatric Inventory–Questionnaire (NPI-Q) [43]	A brief assessment of neuropsychiatric symptoms	x	x				
Cornell Scale for Depression in Dementia (CSDD) [44]	Depression in persons with dementia.	x	x	x	x	x	x
Confusion Assessment Method (CAM) [45]	Assesses the occurrence of delirium	x	x		x		
Functioning in daily living and physical symptoms							
Physical Self-Maintenance Scale (PSMS) [46]	Measures basal ADL	x	x	x	x	x	x
Life-Space Assessment (LSA) [47]	Assessment of life-space mobility		x	x	x	x	x
Short Physical Performance Battery (SPPB) [48]	Chair stand, balance, and walking		x	x	x	x	x
Quality of life							
Quality of Life in Alzheimer’s Disease – patient rated (QoL-AD) [49]	Measures disease-specific QoL	x					
Quality of Life in Alzheimer’s Disease – staff rated (QoL-AD) [49]	Measures disease-specific QoL	x					
Quality of Life in Alzheimer’s Disease – patient or staff rated (QoL-AD) [49]	Measures disease-specific QoL		x		x		
Quality of Life in Late-Stage Dementia (QUALID) [50, 51]	Measures QoL in severe dementia	x	x	x	x	x	x
EQ-5D (including VAS) [52]	Measures health-related QoL	x	x	x	x	x	x
Medication regular prescription	Drug type and daily dose	x	x	x	x	x	x
Cost of care							
Resource Utilization in Dementia (RUD) [54]	Formal and informal care	x	x	x			
Resource Utilization in Dementia – Formal Care (RUD-FOCA) [55]	Measures direct care time required in nursing				x	x	x
Caregiver burden							
Relative Stress Scale (RSS) [56]	Assessment of caregiver burden	x					

Due to collaboration with other research groups and input from research assistants in the field, changes in the baseline dataset were done during the inclusion period

^a^Through collaboration with two research groups interested in a) palliative care and b) physical strength, three assessment tools for palliative care and two physical tests were added to the baseline-dataset after 153 patients had been included

^b^After the inclusion of 544 patients, some assessment tools were removed from the baseline-data set since the included patients ensured sufficient power to complete the planned analysis, and to keep the dataset at a minimum to ensure that the patients and their caregivers were not exhausted by the large size of the dataset

The data collected at baseline and follow-up are summarised in Table 1. Due to collaboration with other research groups and input from research assistants in the field, changes in the baseline dataset were implemented during the inclusion period. Some assessment tools were removed because they were too demanding for the patients to complete and/or for the NH staff to implement. Other assessment tools were added to the baseline data collection due to input from other researchers.

### Measures

#### Cognitive function and severity of dementia

The Mini Mental Status Examination (MMSE), ranging from 0-30, and the eight-question version of Severe Impairment Battery (SIB-8), ranging from 0-16, were used to assess cognitive functioning. A higher score indicates better cognitive function on both scales [23, 24]. Changes in ADL and cognitive functioning over the last 10 years were assessed with the Informant Questionnaire of Cognitive Decline in the Elderly (IQCODE), a proxy-based scale with 16 items scored 1-5 [25, 26]. A mean score of 3.44 and above indicates dementia [27]. Detailed clinical information on debut, course, and symptoms of the dementia were collected based on a structured questionnaire.

Dementia severity was assessed with the Clinical Dementia Rating Scale (CDR), a global rating scale covering six domains of cognitive and functional performance [28]. The CDR can be scored according to an algorithm, giving a total score of 0 (no cognitive impairment), 0.5 (mild cognitive impairment), 1 (mild dementia), 2 (moderate dementia), 3 (severe dementia); however, CDR can also be scored by the CDR sum of boxes (CDR-SOB), ranging from 0 to 18, where a higher score indicates more severe dementia [29]. The two scoring systems intercorrelate highly with kappa scores ranging between 0.86 and 0.94 and a 93% overall correct classification rate [29].

The Functional Assessment Staging of Alzheimer Disease (FAST) scale, ranging from 0-7 with a higher score defining lower function, was used to give a more detailed assessment at the severe stage of dementia [30].

Based on all available information, no cognitive impairment, mild cognitive impairment (MCI) and dementia, as well as dementia subtypes were independently diagnosed by two of the authors (G.S. and S.B.), one psychiatrist and one intern specialising in psychiatry, both of whom were experienced old age psychiatrists and researchers, with the possibility of consulting a third specialist, also a psychiatrist (K.E.) to reach a consensus. Dementia was diagnosed according to the international classification of diseases, version 10, research criteria (ICD-10) [22], and MCI was diagnosed according to Winblad’s criteria [21]. Alzheimer’s disease, vascular dementia, and mixed AD/VaD were diagnosed according to the ICD-10 criteria [22]; Lewy body dementia was diagnosed according to the DLB consortium criteria [19]; and Frontotemporal dementia was diagnosed according to the Manchester-Lund criteria [20].

### Physical health status

Blood pressure, pulse, weight, and height were measured following a standardised procedure. General physical health was assessed using the General Medical Health Rating (GMHR) scale, a one-item, global rating scale with four categories (excellent, good, fair, poor) [31].

Pain was assessed by the Mobilization-Observation-Behaviour-Intensity-Dementia Pain Scale (MOBID-2), consisting of 10 items, each item ranging from 0 to 10, where a higher score indicates more severe pain. Additionally, the overall pain was assessed on a 10-point visual analogue scale (VAS) [32].

Extrapyramidal symptoms were assessed through observations with the six-item version of the Unified Parkinson’s Disease Rating Scale (UPDRS-6), ranging from 0-24, where a higher score indicates more severe symptoms [33].

Physical symptoms were assessed with the Edmonton symptom assessment system (ESAS), ranging from 0-10, where a higher score indicates more severe symptoms [34]. Overall functioning was assessed with the Karnofsky performance status scale (KPS), an 11-step rating scale from normal functioning (100) to dead (0) [35]. Two subscales from the Residents Assessment Instrument (RAI-NH) were used to evaluate the patients’ skin and nutrition condition [36].

For assessment of comorbidity, the Charlson’s comorbidity index, with 18 different groups of diseases, was used [37].

### Neuropsychiatric and depressive symptoms

Neuropsychiatric symptoms (NPS) were assessed using the Neuropsychiatric Inventory 12-item nursing home version (NPI-NH) [38, 39]. Severity (score 1 -3) was multiplied by frequency (score 1 -4), giving an item score ranging from 0-12, where a higher score indicates more severe symptoms. A clinically significant NPS (CS-NPS) was defined as an NPI item score of four and above [40].

NPI sub-syndrome scores were calculated based on a previous principal component analysis: NPI agitation (agitation/aggression, disinhibition, and irritability), NPI psychosis (delusions and hallucinations), and NPI affective (depression and anxiety) [41, 42]. The brief Neuropsychiatric Inventory–Questionnaire (NPI-Q) was completed at baseline by the patient’s next of kin in order to assess NPS symptoms from the debut of dementia and prior to nursing home admission [43].

Depressive symptoms were assessed with the Cornell scale for depression in dementia (CSDD), a 19-item scale (0-2 points) ranging from 0-38, where a higher score indicates more severe symptoms [44]. To detect delirium, the Confusion Assessment Method (CAM), a four-step algorithm assessing delirium symptoms, was performed [45].

### Functioning in daily living and physical symptoms

Functional status was assessed with the Physical Self-Maintenance Scale (PSMS), a six-item scale (scored 1-5) ranging from 6-30, where a higher score indicates lower level of functioning [46]. The Life-Space Assessment (LSA) was performed to assess the range, independence, and frequency of the patient’s movement over the last two weeks [47]. Balance and gait speed were assessed with the Short Physical Performance Battery (SPPB), ranging from 0-12, where a higher score indicates better physical performance [48].

### Quality of life

Quality of life (QoL) was assessed with the Quality of Life in Alzheimer’s Disease (QoL-AD) scale; 13 items rated from 1 to 4 (range 13-52), with a higher score indicating a better QoL [49]. The QoL-AD was completed by both the patient and the caregiver, when possible.

The Quality of Life in Late-Stage Dementia scale (QUALID) is a proxy-based assessment scale consisting of 11 items with scores from 1 to 5, ranging from 11-55, with lower scores indicating a better QoL [50, 51].

The EQ-5D is a brief five-dimension self-reported instrument for generic health status (mobility, self-care, usual activities, pain/discomfort, and anxiety/depression), scored 0-2, with a sum score ranging from 0-10 and a lower score indicating better functioning. The EQ-5D includes a visual analogue scale ranging from 0 (worst imaginable health state) to 100 (best imaginable health state) [52].

### Medication

Regular medication use from admission to the nursing home and onward was recorded from the patients’ medical records using the Anatomic Therapeutic Chemical (ATC) classification system and defined daily doses (DDD) [53]. Psychotropic medications were grouped as: antipsychotics (N05A except lithium), antidepressants (N06A), anxiolytics (N05B), hypnotic/sedatives (N05C), and anti-dementia medication (N06D).

### Cost of care

The use of formal and informal care the last month before admission to the NH was assessed with the Resource Utilization in Dementia Questionnaire (RUD) [54]. To assess formal care after admission to the NHs, the Resource Utilization in Dementia – Formal Care (RUD-FOCA) was used at the follow-up assessments [55].

### Caregiver burden

To assess caregiver burden during the last month before the patients’ admission to the NH, the Relative Stress Scale (RSS), a 15-item scale scored from 0 to 4, where a higher score denotes a higher burden, was used [56, 57].

### Linkage to registry and databases

Data can be linked to the Norwegian Prescription Database (NorPD), containing data about dispensed drugs in Norway; the Norwegian Patient Register (NPR), which contains information on all patients referred to or having received treatment in the specialist health services; the IPLOS register, a Norwegian statutory health register for municipal health services; The Cancer Registry of Norway, containing information about all cancer cases in Norway; and the Cause of Death Registry.

### Ethical and legal considerations

The patients’ capacity to consent to participation in the study was considered by the NH staff, including the physician. Written consent for participation was obtained from all participants with the capacity to consent. For participants lacking the capacity to consent, their next of kin gave consent on behalf of the patients. The next of kin gave written consent for their own participation in the study, as they provided information about themselves. The Regional Ethics Committee for Medical Research in South-Eastern Norway approved the study (2011/1738a).

## Results

Of the 696 included patients, 2.4% had no cognitive impairment, 13.8% had mild cognitive impairment, and 83.8% had dementia. Twelve persons were under the age of 65, 10 of whom (83.3%) had dementia. Saliva samples for DNA testing were taken from 611 patients (87.7%). Table 2 presents demographic characteristics and level of functioning at baseline for the total cohort and for participants with and without dementia. The patients with dementia were younger (*p* = 0.002), more often married or had partners (vs. unmarried, divorced, or widowed) (*p* = 0.015), had better physical health (*p* = 0.013), and few had very impaired vision (vs. mildly impaired or normal vision) compared to patients without dementia (*p* = 0.036). Sedatives were more often prescribed to the patients without dementia than to patients with dementia (*p* = 0.004), and anti-dementia medications were more often prescribed to patients with dementia (*p* < 0.001). Patients without dementia had more pain than patients with dementia (*p* < 0.001). Patients without dementia scored higher on self-rated scores quality of life assessments, both on the QoL-AD (*p* = 0.025) and the EQ-5D (*p* < 0.001), while patients with dementia scored higher on all the overall QoL VAS scales, both patient-rated (*p* = 0.018) and staff-rated (*p* = 0.023).

**Table 2:** Demographic and clinical data of the patients at admission to nursing homes (NH)

	All patients	Patients with dementia	Patients without dementia	p-value*
	*n*= 696	*n*= 583	*n*= 113	
Age mean (SD)	84.5 (7.5)	84.1 (7.5)	86.5 (7.0)	0.002^a)^
Female gender	445 (63.9)	375 (64.3)	70 (61.9)	0.630^b)^
Unmarried/divorced/widowed vs. married/partner n/N	478/687 (69.6)	390/576 (67.6)	88/111 (79.3)	0.015^b)^
	*n*=516	*n*=428	*n*=88	
Education in years – mean (SD)	8.34 (2.8)	8.30 (2.9)	8.50 (2.4)	0.549^a)^
Residence before admission	*n*=520	*n*=428	*n*=92	
Private home	230 (44.2)	194 (45.3)	36 (39.1)	0.278^b)^
Sheltered flat	71 (13.7)	59 (13.8)	12 (13.0)	0.851^b)^
Care Home (CH)	5 (1.0)	5 (1.2)	-	0.592^c)^
CH with Nursing	134 (25.8)	110 (25.7)	24 (26.1)	0.939^b)^
Hospital	78 (15.0)	58 (13.6)	20 (21.7)	0.046^b)^
Other	2 (0.4)	2 (0.5)	-	1.000^c)^
Type of unit	*n*= 696	*n*= 583	*n*= 113	
Regular unit (RU)	385 (55.3)	303 (52.0)	82 (72.6)	<0.001^b)^
Respite and rehabilitation unit (RRU)	85 (12.2)	64 (11.0)	21 (18.6)	0.024^b)^
Special care unit (SCU)	226 (32.5)	216 (37.0)	10 (8.8)	<0.001^b)^
GMHR	*n*=666	*n*=557	*n*=109	
GMHR dichotomized; poor/fair	349 (52.4)	280 (50.3)	69 (63.3)	0.013^b)^
MOBID-2				
Total score	*n*=667	*n*=557	*n*=110	
mean (SD)	2.1 (2.2)	1.96 (2.1)	2.84 (2.4)	<0.001^a)^
Overall pain at a 10-point scale	*n*=597	*n*=490	*n*=107	
mean (SD)	2.4 (2.5)	2.17 (2.4)	3.42 (2.8)	<0.001^a)^
UPDRS-6	*n*=528	*n*= 446	*n*=82	
mean (SD)	3.6 (3.7)	3.6 (3.7)	3.6 (3.4)	0.870^a)^
Vision	*n*=681	*n*=569	*n*=112	
Normal	161 (23.6)	138 (24.3)	23 (20.5)	0.036^b)^
Mildly impaired	431 (63.3)	365 (64.1)	66 (58.9)	
Very impaired	89 (13.1)	66 (11.6)	23 (20.5)	
Hearing	*n*=682	*n*=571	*n*=111	
Normal	299 (43.8)	259 (45.4)	40 (36.0)	0.193^b)^
Mildly impaired	290 (42.5)	236 (41.3)	54 (48.6)	
Very impaired	93 (13.6)	76 (13.3)	17 (15.3)	
Use of psychotropic medication**	*n*= 696	*n*= 583	*n*= 113	
Antipsychotics	84 (12.1)	72 (12.4)	12 (10.6)	0.605^b)^
Antidepressants	199 (28.6)	167 (28.6)	32 (28.3)	0.944^b)^
Anxiolytics	108 (15.5)	89 (15.3)	19 (16.8)	0.677^b)^
Sedatives	167 (23.9)	128 (21.9)	39 (34.5)	0.004^b)^
Anti-dementia drugs	169 (24.3)	163 (28.0)	6 (5.3)	<0.001^b)^
CSDD	*n*=657	*n*=548	*n*=109	
mean (SD)	6.4 (5.2)	6.6 (5.3)	5.7 (4.7)	0.094^a)^
PSMS	*n*=694	*n*=582	*n*=112	
mean (SD)	15.3 (4.5)	15.3 (4.5)	15.4 (4.7)	0.797^a)^
QoL-AD***				
Patient rated	*n*=276	*n*=227	*n*=49	
mean (SD)	33.1 (5.5)	32.7 (5.4)	34.7 (5.6)	0.025^a)^
Staff rated	n=346	n=300	n=46	
mean (SD)	31.8 (5.8)	31.7 (5.7)	32.6 (6.8)	0.327^a)^
QUALID	*n*=691	*n*=579	*n*=112	
mean (SD)	20.0 (7.2)	20.0 (7.2)	19.4 (7.1)	0.402^a)^
EQ-5D				
Patient rated	*n*=219	*n*=172	*n*=47	
mean (SD)	3.3 (2.2)	2.9 (2.1)	4.7 (2.3)	<0.001^a)^
Staff rated	*n*=455	*n*=392	*n*=63	
mean (SD)	5.3 (1.7)	5.3 (1.7)	5.5 (1.9)	0.393^a)^
Overall QoL VAS-scale	n=520	n=421	n=99	
mean (SD)	60.4 (23.5)	62.1 (23.1)	53.1 (23.7)	0.001^a)^
Patient rated	*n*=197	*n*=153	*n*=44	
mean (SD)	61.6 (23.5)	63.7 (23.2)	54.2 (23.4)	0.018^a)^
Staff rated	*n*=314	*n*=260	*n*=54	
mean (SD)	59.2 (23.2)	60.6 (22.9)	52.7 (24.0)	0.023^a)^

All figures in (%) if not otherwise stated

*SD* standard deviation

*GMHR* General Medical Health Rating Scale

*MOBID-2* Mobilization-Observation-Behaviour-Intensity-Dementia Pain Scale

*UPDRS-6* Unified Parkinson’s Disease Rating Scale, six-item version

*CSDD* Cornell scale for depression in dementia

*PSMS* Physical Self-Maintenance Scale

*QoL-AD* Quality of Life Alzheimer Disease

*QUALID* Quality of Life in Late Stage Dementia

*EQ-5D* a standardised instrument for use as a measure of health outcome

*QoL* Quality of Life

*VAS* Visual Analogue Scale

*p-value for difference in patients with and without dementia

**Psychotropic medications were grouped as: antipsychotics (N05A except lithium), antidepressants (N06A), anxiolytics (N05B), hypnotic/sedatives (N05C), and anti-dementia medication (N06D)

***In this sample, 132 patients had their QoL-AD score assessed by both themselves and staff:

Patient scored: mean 31.5 (4.9)

Staff scored: mean 34.0 (5.6)

*p*-value 0.000^a)^

^a)^ Independent Student’s t-test

^b)^ Pearson Chi-square Test

^c)^ Fisher’s Exact Test

^d)^ Mann-Whitney U Test

At the 18-month follow-up, 371 of 696 patients were still in the study. However, 22 were not assessed at the 18-month follow-up, and 324 left the study: 261 due to death and 63 due to other reasons. A summary of the number of participants at each assessment is given in Table 3. Differences in age, sex, cognition, and physical health between remaining patients and those lost to follow-up are described in Table 4.

**Table 3:** Number of participants at each assessment in the REDIC-NH cohort

	Baseline	6.month	12.month	18.month
Number included	696	543	446	372
Number assessed	696	508	427	349
Number that left the study		153	250	324
- Due to death		115	191	261
- Due to other reasons		38	59	63
- NH withdrawn		2	2	3
- Patient withdrawn		4	8	9
- Moved to another unit or NH		15	21	23
- Moved home		17	28	28

**Table 4:** Difference in baseline assessments between patients participating at 18-month assessment vs. lost to follow-up

	Still participating after 18-months	Lost to follow-up before 18-months			
		Due to death	*p*-value*	Due to other reasons	*p*-value**
Age n - year (SD)	372 - 83.7 (7.9)	261 - 86.2 (6.4)	<0.001^a)^	63 - 82.2 (8.0)	0.173^a)^
Women n/N - %	252/372 - 67.7%	151/261 - 57.9%	0.011^b)^	43/63 - 68.3%	0.936^b)^
CDR-SOB n - mean score (SD)	369 - 10.2 (4.1)	257 - 10.7 (4.6)	0.108^a)^	61 - 8.9 (4.0)	0.026^a)^
GMHR n/N - % dichotomized; poor/fair	154/357 - 43.1%	160/250 - 64.0%	<0.001^b)^	35/59 - 59.3%	0.021^b)^

*SD* Standard deviation

*CDR-SOB* Clinical Dementia Rating Scale sum of boxes

*GMHR* General Medical Health Rating Scale

*p-value for difference between patients participating at 18-month follow up vs. lost to follow-up due to death

**p-value for difference between patients participating at 18-month follow-up vs. lost to follow-up due to all other reasons

^a)^ Independent Student’s t-test

^b)^ Pearson Chi-square Test

To compare the age and sex of included vs. excluded patients, 38 out of the 47 NHs collected data on the gender and age of all residents eligible for inclusion. Of 1331 eligible patients in these 38 NHs, 607 were included and 724 were excluded (205 declined inclusion, 191 died before inclusion took place, and 328 for reasons not known). The mean age of participants was 84.5 years (SD 7.5), while for non-participants it was 83.6 years (SD 9.3) (independent student *t*-test, *p* = 0.048); 64.4% of participants were women, while 56.6% of non-participants were women (Chi-square test, *p* = 0.004).

Table 5 presents dementia diagnoses and scores on cognitive tests at baseline. The MMSE mean score was higher for patients without dementia than for patients with dementia. Alzheimer's disease was the most prevalent dementia diagnosis. Only 55.9% of the patients with dementia had a diagnosis of dementia registered in their nursing home records.

**Table 5:** Cognition and prevalence of dementia at admission to nursing homes (NH)

		All patients *n*=696	Patients with dementia *n*=583	Patients without dementia *n*=113	*p*-value*
Prevalence of Dementia^1^	No dementia	17 (2.4)			
	Mild Cognitive Impairment	96 (13.8)			
	Dementia	583 (83.8)			
Dementia sub-types^1^	Alzheimer disease (AD)		414 (71.0)		
	Vascular Dementia (VaD)		46 (7.9)		
	Mixed AD/VaD		11 (1.9)		
	Frontotemporal Dementia		47 (8.1)		
	Lewy Body Dementia		22 (3.7)		
	Other		43 (7.4)		
Dementia diagnosis according to NH-records			326 (55.9)	8 (7.1)	<0.001^b)^
Cognition	MMSE sum (n) mean (SD)	(611) 16.0 (6.3)	(511) 14.7 (5.5)	(100) 22.6 (5.6)	<0.001^a)^
	CDR-SOB (n) mean (SD)	(687) 10.3 (4.3)	(576) 11.2 (3.6)	(111) 5.3 (4.2)	<0.001^a)^
	SIB-8 sum (n) mean (SD)	(601) 12.2 (3.7)	(502) 11.8 (3.8)	(99) 14.6 (2.7)	<0.001^a)^
	IQCODE score > 3.44	121 (17.4)	115 (95)	6 (5)	<0.001^b)^
	FAST value ≥ 4 n/N	472/540 (87.4)	449/434 (96.7)	91/38 (41.8)	<0.001^b)^

All figures in (%) if not otherwise stated

*MMSE* Mini-Mental-State-Examination

*SD* standard deviation

*CDR-SOB* Clinical Dementia Rating Scale sum of boxes

*SIB-8* Severe Impairment Battery, the eight-question version

*IQCODE* Informant Questionnaire of Cognitive Decline in the Elderly

*FAST* Functional Assessment Staging of Alzheimer Disease

*p-value for difference between patients with and without dementia

^1^ Assessed by two experienced researchers/clinicians independently based on all given information

^a)^ Independent Student’s t-test

^b)^ Pearson Chi-square Test

^c)^ Fisher’s Exact Test

Table 6 presents the prevalence of NPS at baseline. Of the patients with dementia, 62.9% had at least one clinically significant NPS, vs. 48.6% of the patients without dementia (*p* = 0.005). Anxiety and depression were the most prevalent NPS. Patients with dementia had a higher NPI-12 sum-score, compared to patients without dementia (sum score 10.0 vs. 4.0, *t*-test *p* < 0.001). Anxiety, depression, and irritability were the most prevalent NPS among the patients with dementia. Patients with dementia more often experienced delusions, hallucinations, agitation, anxiety, disinhibition, irritability, and aberrant motor behaviour compared to patients without dementia.

**Table 6:** Clinically significant neuropsychiatric symptoms at admission to nursing homes (NH)

Prevalence of CS-NPS	All patients Total *N*=696	Patients with dementia Total *N*=583	Patients without dementia Total *N*=113	*p-value*
	n/N	n/N	n/N	
Delusions	97/686 (14.1)	90/575 (15.7)	7/111 (6.3)	0.010^a)^
Hallucinations	34/688 (4.9)	33/576 (5.7)	1/112 (0.9)	0.031^a)^
Agitation	99/689 (14.4)	93/577 (16.1)	6/112 (5.4)	0.003^a)^
Depression	148/688 (21.5)	125/576 (21.7)	23/112 (20.5)	0.784^a)^
Anxiety	141/690 (20.4)	126/578 (21.8)	15/112 (13.4)	0.043^a)^
Euphoria	23/687 (3.3)	21/567 (3.6)	2/111 (1.8)	0.562^b)^
Apathy	109/687 (15.9)	95/575 (16.5)	14/112 (12.5)	0.287^a)^
Disinhibition	101/687 (14.7)	92/575 (16.0)	9/112 (8.0)	0.029^a)^
Irritability	122/684 (17.8)	110/572 (19.2)	12/112 (10.7)	0.031^a)^
Aberrant Motor Behaviour	73/687 (10.6)	69/575 (12.0)	4/112 (3.6)	0.008^a)^
Night-time Behaviour	112/689 (16.3)	98/577 (17.0)	14/112 (12.5)	0.239^a)^
Eating Change	71/688 (10.3)	58/576 (10.1)	13/112 (11.6)	0.625^a)^
Any symptom	413/682 (60.6)	359/571 (62.9)	54/111 (48.6)	0.005^a)^
NPI 12 sum median (range)	*n*=693 8.0 (0 - 123)	*n*=581 10.0 (0 - 123)	*n*=112 4.0 (0 - 66)	<0.001^c)^
NPI-AGITATION median (range)	*n*=678 1.0 (0 - 36)	*n*=566 1.0 (0 - 36)	*n*=112 0.0 (0 - 36)	<0.001^c)^
NPI-PSYCHOSIS median (range)	*n*=683 0.0 (0 - 24)	*n*=572 0.0 (0 - 24)	*n*=111 0.0 (0 - 12)	<0.001^c)^
NPI-AFFECTIVE median (range)	*n*=687 1.0 (0 - 24)	*n*=575 1.0 (0 - 24)	*n*=112 0.5 (0 - 24)	0. 125^c)^

All figures in (%) if not otherwise stated

CS-NPS - clinically significant NPS, defined as an NPI sub-symptom of 4 and above

NPI 12 sum - Neuropsychiatric Inventory sum of 12 items

NPI-AGITATION sum of agitation/aggression, disinhibition, and irritability

NPI-PSYCHOSIS sum of delusion and hallucination

NPI-AFFECTIVE sum of depression and anxiety

^a)^ Pearson Chi-square test

^b)^ Fisher’s Exact Test

^c)^ Mann-Whitney U test

The most common comorbidity diseases, according to the Charlson’s comorbidity index, in both patients with and without dementia were cardiovascular diseases (coronary diseases, congestive heart failure, and cerebrovascular disease), diabetes, and cancer (see Table 7). Patients without dementia more often had cardiovascular diseases (coronary diseases (*p* = 0.009), congestive heart failure (*p* = 0.009), pulmonary disease (*p* = 0.018), connective tissue disease (*p* = 0.013), diabetes with complications (*p* = 0.001), hemiplegia/paraplegia (*p* = 0.003), and renal disease (*p* = 0.002), while patients with dementia more often had dementia (*p* < 0.001), according to the Charlson’s comorbidity index. Nevertheless, only 80.6% of patients diagnosed with dementia in the study had dementia according to the Charlson’s comorbidity index, while 20.6% of the patients not diagnosed with dementia in the study had a diagnosis of dementia according to the Charlson’s comorbidity index.

**Table 7:** Prevalence of diseases according to Charlson Comorbidity Index at admission to nursing homes (NH)

	All patients	Patients with dementia	Patients without dementia	*p-value*
Coronary disease	167/664 (25.2)	130/559 (23.3)	37/105 (35.2)	0.009^a)^
Acute myocardial infarction	97/664 (14.6)	77/558 (13.8)	20/106 (18.9)	0.176^a)^
Congestive heart failure	137/654 (20.9)	105/549 (19.1)	32/105 (30.5)	0.009^a)^
Peripheral vascular disease	91/656 (13.9)	75/551 (13.6)	16/105 (15.2)	0.659^a)^
Cerebrovascular disease	161/664 (24.2)	135/556 (24.3)	26/108 (24.1)	0.963^a)^
Dementia	467/659 (70.9)	445/552 (80.6)	22/107 (20.6)	<0.001^a)^
Pulmonary disease	82/666 (12.3)	61/556 (11.0)	21/110 (19.1)	0.018^a)^
Connective tissue disease	58/664 (8.7)	42/557 (7.5)	16/107 (15.0)	0.013^a)^
Peptic ulcer disease	58/667 (8.7)	44/558 (7.9)	14/109 (12.8)	0.093^a)^
Liver disease	3/673 (0.4)	3/564 (0.5)	0	1.000^b)^
Diabetes	101/676 (14.9)	79/566 (14.0)	22/110 (20.0)	0.104^a)^
Diabetes with complications	25/676 (3.7)	14/564 (2.5)	11/110 (10.0)	0.001^b)^
Hemiplegia or paraplegia	21/664 (3.2)	12/555 (2.2)	9/109 (8.3)	0.003^b)^
Renal disease	86/670 (12.8)	62/560 (11.1)	24/110 (21.8)	0.002^a)^
Cancer	98/671 (14.6)	77/563 (13.7)	21/108 (19.4)	0.120^a)^
Metastatic cancer	17/667 (2.5)	12/559 (2.1)	5/108 (4.6)	0.172^b)^
Severe liver disease	3/674 (0.4)	3/565 (0.5)	0	1.000^b)^
HIV disease	1/668 (0.1)	1/558 (0.2)	0	1.000^b)^

All figures in (%)

^a)^ Pearson Chi-square test

^b)^ Fisher’s Exact Test

## Discussion

The present study is the first Norwegian study and one of few international studies following long-term NH patients from admission to the NH and until death or up to 36 months, assessed regularly with standardised assessment tools.

The main finding of this study is the high prevalence of dementia (83.8%) at admission to the NH, comparable to figures in cross-sectional studies of Norwegian NHs showing a prevalence of 81.5% [3] and 78.5% [6], but different from a descriptive study from Belgium [7] in which 48% had dementia at admission. The patients with dementia at admission were younger, had better physical health, less pain, and better vision than patients without dementia, indicating that they were not admitted to the NH for physical health problems, but for their dementia. Furthermore, 62.9% of the patients with dementia had at least one clinically significant NPS, where anxiety and depression were most prevalent. A review by Selbæk et al. reported a 82% prevalence of at least one clinically significant NPS in patients with dementia living in nursing homes, and although the prevalence of individual symptoms varied, the highest prevalence figures were found for agitation and apathy [4]. Two longitudinal NH studies reported that NPS in patients with dementia are common and that individual NPS have a fluctuating course. A Norwegian study reported irritability, agitation, and disinhibition to be most prevalent in patients with dementia [5], and a study from the Netherlands reported apathy, depression, and aberrant motor behaviour to be the most frequent NPS [58].

Of the 83.8% participants with dementia, according to the two experienced psychiatrists, only 55.9% had a dementia diagnosis documented in their records, and 80.6% had dementia according to the Charlson’s comorbidity index. Of the patients without a dementia diagnosis, according to the two experienced psychiatrists, 7.1% had a dementia diagnosis documented in their records, and 20.6% had dementia according to the Charlson’s comorbidity index. All cognitive measures showed significantly lower scores for persons with dementia compared to those without dementia. However, persons without dementia also scored quite low on the cognitive scales, especially on the MMSE (mean 22.6), and a large proportion had a FAST score ≥ 4 (41.8%), see Table 5. The discrepancy between the prevalence of dementia and a dementia diagnosis in the patient records in this study are in line with several other studies, both previous Norwegian [3] and international studies [59–62], and could be explained by the lack of clinical examination of the patients in the study as well as nursing home doctors underdiagnosing dementia. In addition, the discrepancy between dementia diagnoses set by the researcher and documentation in NH records can be due to various definitions and diagnostic criteria for dementia. Physicians in the municipality and in the NHs mainly use the International Classification of Primary Care second version (ICPC-2) [63], whereas researchers use other criteria, such as the international classification of diseases, version 10 (ICD-10 criteria), Winblad’s criteria, the DLB consortium criteria, and the Manchester-Lund criteria [19–22]. Physicians are not constantly present in nursing homes, and resources for dementia diagnostics in primary care are scarce; hence, diagnostics are often superficial and performed rapidly [64]. Another explanation for the discrepancy can be that the diagnosis of dementia is not given priority in nursing homes, as it is often claimed that there is no curative treatment for dementia and the diagnosis does not benefit the patient. However, a lot of other interventions for preventing functional decline and improving the quality of life for persons with dementia can be performed [65, 66] if patients are diagnosed adequately.

QoL was assessed with three different assessment scales: QoL-AD, QUALID, and EQ-5D, and the results differed considerably between the different scales. Assessed with the QoL-AD, rated by the patients themselves, patients without dementia had better QoL than patients with dementia. When patients rated themselves with EQ-5D (including VAS), the patients with dementia reported better QoL than patients without dementia. Regarding QUALID scored by proxy, there was no difference between persons with or without dementia. The disagreement between these scales may be due to the difference between the scales. QoL-AD measures the domains of physical condition, mood, memory, functional abilities, interpersonal relationships, ability to participate in meaningful activities, financial situation, global assessments of self as a whole, and QoL as a whole, and is filled out by the patients, caregivers, or both, while QUALID is a proxy-report instrument that measures 11 observable behaviours about activity and emotional states over the last seven days. The EQ-5D focuses on generic health status, such as specific problems with performing specific physical tasks as mobility, self-care, and usual activities, and whether the patient experiences pain or discomfort, or is anxious or depressed. QoL-AD and QUALID are both designed specifically to measure QoL in persons with dementia, while the EQ-5D is a standardised instrument for use as a measure of health outcomes applicable to a variety of different illnesses and treatments. The disagreement between different assessment methods for QoL, and the difficulties in conceptualising QoL, should lead to caution in interpreting the results.

Compared to a Finnish study from 2011, reporting anti-dementia drug use in 66.8% of persons with dementia [67], the prescription rate of anti-dementia medication in this study seems to be low. But, the result is in line with cross-sectional studies from Norway and Sweden done in 2004/2005, 2007 and 20110/2011, reporting a prevalence range from 11.3% to 18% [68, 69]. That only 55.9% of the participants with dementia according to the two experienced psychiatrists, had a dementia diagnosis documented in their records, can be an explanation for the low anti-dementia medication rate. In addition, the dementia was severe at time for admission to the NH, and anti-dementia medication may have been discontinued due to lack of effect, side effects or polypharmacy.

### Strengths and weaknesses

The longitudinal design with a broad assessment and inclusion of the patients at admission to the NH is a strength of the study. This will allow researchers to analyse and present associations between different patient characteristics and symptoms, and to analyse the stability of these variables over time. It will also be possible to analyse how different baseline characteristics are associated with the course of dementia, in addition to implementing analyses with time-dependent variables. The use of standardised assessment tools – widely used both clinically and in research – will make it possible to compare findings from studies based on this dataset with other studies, both in Norway and internationally.

Furthermore, the high number of participants recruited from different NHs in a large geographical area, covering both urban and rural areas, was a strength of the study. Data were registered using standard and validated assessment tools, covering a broad range of symptoms and topics. The broad data collection allowed researchers to diagnose dementia according to international criteria without a clinical examination of the patients. Even with broad data collection at regular intervals in 696 patients, this study had relatively few missing data. The collection of DNA in a sub-sample of 611 patients is another strength of the study.

Furthermore, data collected in the study can be linked to the unique personal identification numbers of the participants, enabling linkage of data for each patient to five health registers: the Norwegian Prescription Database (NorPD), containing data about dispensed drugs in Norway; the Norwegian Patient Register (NPR), which contains information for all patients referred to or having received treatment in the specialist health services; the IPLOS-register, a Norwegian statutory health register for municipal health services; The Cancer Registry of Norway, containing information about all cancer cases in Norway; and the Cause of Death Registry.

One limitation to the study was that the participants might not have been representative of all patients at admission to NHs, because respite care patients were excluded. Another limitation was that only 38 of the 47 NHs collected data about the patients who were eligible for inclusion, but did not participate, and less than half of the residents eligible for inclusion in these 38 nursing homes were included in the study. There were also more women in the included sample compared to those who did not participate (64.4% vs. 56.6%, Chi-square test *p* = 0.004). These factors may have influenced the representativeness of the sample. From baseline to 18 months, 324 (46.5%) of the participants dropped out of the study, 261 of them (80.5%) dropped out due to death. Only nine (2.7%) of the participants who dropped out before the 12-month assessment withdrew their consent, indicating that the high dropout rate probably did not bias the representativeness significantly. A high number of NH staff participated in the data collection. Even though they had participated in a training program, this could be a limitation to the study.

The statistical differences between persons with and without dementia in some variables were, in this paper, descriptively presented, and these results should be interpreted with caution.

## Conclusions

In this paper, we describe the methods of our study in detail and our cohort’s baseline demographic characteristics. The prevalence rates of dementia and NPS reported in this study could contribute to a greater understanding of the needs of nursing home patients and, thus, increase the knowledge in order to improve the quality of care for nursing home residents. In addition, the findings could be valuable to stakeholders and organisations when planning nursing home care for these patients.
